# *Syzygium aromaticum* ethanol extract reduces high-fat diet-induced obesity in mice through downregulation of adipogenic and lipogenic gene expression

**DOI:** 10.3892/etm.2012.609

**Published:** 2012-06-13

**Authors:** CHANG HWA JUNG, JIYUN AHN, TAE-IL JEON, TAE WAN KIM, TAE YOUL HA

**Affiliations:** 1 Division of Metabolism and Functionality Research, Korea Food Research Institute, Seongnam 463-746;; 2 Department of Food Science and Biotechnology, Andong National University, Gyeongbuk 760-749, Republic of Korea

**Keywords:** obesity, lipogenesis adipogenesis, *Syzygium aromaticum*

## Abstract

Numerous medicinal plants and their derivatives have been reported to prevent obesity and related diseases. Although *Syzygium aromaticum* has traditionally been used as an anodyne, carminative and anthelmintic in Asian countries, its potential in the prevention and treatment of obesity has not yet been explored. Therefore, the present study investigated the anti-obesity effect of *S. aromaticum* ethanol extract (SAE) both *in vitro* and *in vivo*. To evaluate the anti-obesity potential of SAE *in vitro*, the effect of SAE treatment on adipocyte differentiation in 3T3-L1 cells was investigated. To evaluate its potential *in vivo*, mice were assigned to three groups: a group fed the American Institute of Nutrition AIN-76A diet (normal group), an experimental group fed a high-fat diet (HFD group) and an experimental group fed an HFD supplemented with 0.5% (w/w) SAE (HFD + SAE group). After 9 weeks of feeding, the body weight; white adipose tissue (WAT) mass; serum triglyceride (TG), total cholesterol (TC), high-density lipoprotein (HDL) cholesterol, glucose, insulin and leptin; hepatic lipid accumulation; and levels of lipid metabolism-related genes in the liver and WAT were measured. *In vitro* investigation of the effect of SAE treatment on 3T3-L1 cells revealed that it had efficiently inhibited the conversion of cells into adipocytes in a dose-dependent manner. *In vivo* investigation revealed that SAE supplementation had significantly decreased HFD-induced increases in the body weight, liver weight, WAT mass, and serum TG, TC, lipid, glucose, insulin and leptin levels. Consistent with its effects on liver weight and WAT mass, SAE supplementation was found to have suppressed the expression of lipid metabolism-related proteins, including SREBP-1, FAS, CD36 and PPARγ in the liver and WAT, in addition to downregulating mRNA levels of transcription factors including Srebp and Pparg. SAE inhibits fat accumulation in HFD-fed mice via the suppression of transcription factors integral to adipogenesis and lipogenesis, suggesting its potential in preventing obesity.

## Introduction

Overweight and obesity have become serious global problems in recent years, with a recent report estimating that 1.5 billion adults worldwide are overweight, among whom over 200 million men and almost 300 million women are obese (1). Overweight and obesity were once considered problems associated with high-income nations, but their incidence is now increasing in low and middle-income countries, a trend that is attributed to chronic alcoholism, food overconsumption and sedentary lifestyle. Overweight and obese individuals face the risk of numerous chronic diseases, including heart disease, diabetes and some types of cancer, as well as psychological and social problems.

Overweight and obesity, as well as the noncommunicable diseases associated with their development, are largely preventable. Several steps can be taken not only at the individual and societal levels but also at the molecular level to prevent and treat overweight and obesity. At the individual level, individuals are able to limit their consumption of fat and sugar, increase their consumption of fruits and vegetables, and engage in regular physical activity. At the societal level, governments and the food industry can promote healthy diets. At the molecular level, researchers identify and/or develop agents that prevent or treat obesity. A recent research focus has been the development of anti-obesity agents derived from natural products, a process that has several safety and economic advantages over the development of pharmaceuticals via years of investment in drug development. Such research has been bolstered by recent reports that traditional herbal extracts have been found effective in reducing body weight *in vivo* (2,3).

Among these extracts, the dried aromatic flower buds of *Syzygium aromaticum* (L.) Merr. & Perry (family Myrtaceae), commonly known as cloves, have been reported to be useful in treating dental pain, headache and respiratory disorders in Asian countries (4). Phytochemical studies indicate that eugenol, a member of the phenylpropanoid class of chemical compounds, and its derivatives are responsible for the unique aroma of *S. aromaticum,* and it has been found to have antimicrobial, insecticidal, antioxidant, antitumor, anti-inflammatory and anaesthetic properties (5–10). In spite of these findings and reports of its utility in several medicinal applications, *S. aromaticum* has not been investigated for its potential as an anti-obesity agent or food additive. Therefore, the present study investigated the effect of *S. aromaticum* ethanol extract (SAE) on a mouse model in which obesity had been induced by administering a high-fat diet (HFD).

## Materials and methods

### Materials

Anti-PPARγ (sc-7273), SREBP-1 (sc-366) and CD36 (sc-13572) were purchased from Santa Cruz Biotechnology (Santa Cruz, CA, USA). Antibodies against FAS (#3180), and β-actin (#4967) were purchased from Cell Signaling (Danvers, MA, USA). Insulin (I5500), isobutylmethylxanthine (I7018), dexamethasone (D4902) and Oil Red O (O0625) were purchased from Sigma-Aldrich (St. Louis, MO, USA). Serum and tissue triglyceride (TG), total cholesterol (TC) and high-density lipoprotein-cholesterol (HDL-C) kits were purchased from Wako Chemicals (Osaka, Japan). An insulin ELISA kit was obtained from Shibayagi Co. (Gunma, Tokyo, Japan). A leptin ELISA kit was purchased from R&D Systems (Minneapolis, MN, USA).

### Preparation of extract

Dried flower buds of *Syzygium aromaticum* were purchased from the Omni Herb Company (Seoul, South Korea) and were extracted three times with 70% ethanol at room temperature overnight. The extracts were combined and concentrated using a rotary evaporator, and freeze-dried under a vacuum and dissolved in dimethyl sulfoxide (DMSO).

### Cell culture and differentiation

The 3T3-L1 cells were cultured in Dulbecco’s modified Eagle’s medium (DMEM; Invitrogen, Carlsbad, CA, USA) with 1% penicillin-streptomycin and 10% bovine calf serum at 37°C in 5% CO_2_. The cells were seeded at a density of 4×10^5^ cells/well into a 6-well plate. At 2 days post-confluence (day 0), the cells were exposed to an MDI solution containing 0.5 mM of 3-IBMX, 1 *μ*M of dexamethasone and 1 *μ*g/ml of insulin for 2 days in DMEM with 10% fetal bovine serum (FBS). Following induction, cells were switched to a solution containing DMEM medium supplemented with 1% penicillin-streptomycin, 10% FBS and 167 nm insulin for 2 days. The cells were maintained in a postdifferentiation medium (DMEM containing 1% penicillin-streptomycin and 10% FBS) and changed every 2 days. To examine the effects of SAE on the differentiation of preadipocytes into adipocytes, the cells were treated with various concentrations of SAE at 2 days post confluence (day 0).

### Oil Red O staining

For quantification, cells were fixed with 10% neutral formalin for 1 h at room temperature, washed with phosphate-buffered saline (PBS) and then stained for 1 h with 0.5% Oil Red O in 60% isopropanol. After washing with distilled water, the stained cells were observed under a microscope. The stained lipid droplets were then extracted with isopropanol for quantification by measuring its absorbance at 490 nm.

### Animal models

Male C57BL/6J mice aged 4 weeks were obtained from Orient Bio Inc. (Seoul, South Korea) and acclimatized to laboratory conditions consisting of a 12:12-h light-dark cycle, 24°C and 55% humidity for 1 week. At 5 weeks old the mice were then randomly divided into three groups; a group fed the American Institute of Nutrition AIN-76A diet (normal group), a group fed a HFD (HFD group) and a group fed a HFD supplemented with 0.5% (w/w) SAE (HFD + SAE group) for 9 weeks. The experimental diets were prepared by supplementing the basic AIN-76 diet with 20% fat and 0.5% cholesterol (w/w). Body weight and average daily food intake were measured weekly. The care and use of the animals followed our institutional and national guidelines and all experimental procedures were approved by the Korea Food Research Institute Animal Care and Use Committee (KFRI-IACUC, #2010-0011).

### Sample preparation and procedures

At nine weeks after consuming the experimental diets, the mice were subjected to fasting for 12 h and then sacrificed. Blood samples were collected from the abdominal aorta, centrifuged at 1,000 × g for 15 min and stored at −80°C. The measurement of serum TG, TC and HDL-C levels was performed with the appropriate kit. The measurement of serum insulin and leptin concentrations was performed according to the manufacturer’s instructions of the mouse insulin and leptin ELISA kits. Epididymal, perirenal and liver tissues were excised, weighed and stored at −80°C until use. Liver and epididymal fat pads were either fixed in 4% formalin solution and processed for histological examination or snap frozen in liquid nitrogen and stored at −80°C until protein and RNA extraction.

To determine total lipid, TG and TC levels in the liver, the livers of each group were homogenized in 5 ml NaCl. After 20 ml of Folch solution (chloroform:methanol = 2:1) was subsequently added to each homogenate, the resulting solution was incubated at 4°C for 12 h. The samples were centrifuged for 10 min at 1,000 × g before the organic layer was removed and dried using a Speed Vac. The resulting pellet was dissolved in ethyl alcohol containing 25% Triton X-100 and then assayed for TG, TC and HDL-C levels using indicated commercial kits.

### Histological analysis

The liver and epididymal fat pads were fixed in 4% neutral-buffered formalin, embedded in paraffin, sliced at a thickness of 5 *μ*m and stained with hematoxylin and eosin (H&E) stain. The pathological changes were assessed and photographed under an Olympus BX-51 microscope (Olympus, Tokyo, Japan).

### Western blotting

The isolated liver, adipose tissue and cell extracts were prepared in PRO-PREP protein extraction solution (Intron Biotechnology, Gyeonggi, South Korea) using an MP 40 system (MD Biosciences, St. Paul, MN, USA). The extracts were centrifuged at 15,000 rpm for 20 min at 4°C. The supernatants were boiled with a sodium dodecyl sulfate (SDS)-loading buffer, loaded onto Tris-glycine gels, transferred to polyvinylidene fluoride (PVDF) membranes and visualized with a chemiluminescence reagent (Amersham Bioscience, Piscataway, NJ, USA).

### RNA extraction and real-time PCR

Total RNA was isolated from the liver using TRIzol reagent (Invitrogen, Carlsbad, CA, USA) according to the manufacturer’s instructions. After cDNA was prepared from isolated RNA using a Maxime RT premix (oligo dT primer), the obtained cDNA was used as a template for real-time PCR with the Light Cycler 480 system (Roche, Basel, Switzerland). The specific primers were as follows: Fas sense AGAGATCCCGAGACGCTTCT, antisense GCCTGGTAGGCATTCTGTAGT; Srebp sense GCAGCC ACCATCTAGCCTG, antisense CAGCAGTGAGTCTGCCTT GAT; Pparg sense TCGCTGATGCACTGCCTATG, antisense GAGAGGTCCACAGAGCTGATT; Ppara sense AACATC GAGTGTCGAATATGTGG, antisense AGCCGAATAGTT CGCCGAAAG; and β-actin sense AATACCCCAGCCATG TGTGT, antisense ATGGGCACTGTGTGTGACC. The level of mRNA was expressed as the ratio of signal intensity for each gene relative to β-actin.

### Statistical analysis

Results are expressed as the mean ± SE, and the differences among the three groups were calculated using an analysis of multiple ranges and a one-way variance test using GraphPad Prism4 software (San Diego, CA, USA).

## Results

### SAE inhibits cell differentiation of 3T3-L1 preadipocytes

**Figure 1:**
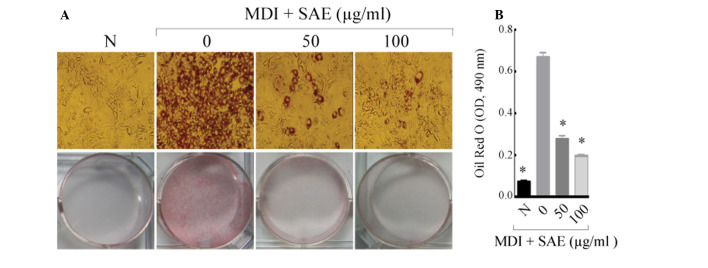
SAE inhibits adipogenic differentiation in 3T3-L1 cells. 3T3-L1 preadipocytes were induced to differentiate into adipocytes by the indicated MDI solution in the presence or absence of SAE. (A) Triglyceride accumulation was determined by Oil Red O staining. (B) Data are expressed as the mean ± SD values of 3 independent experiments. ^*^P<0.01 versus cells exposed to an MDI solution in the absence of SAE. SAE, *S. aromaticum* ethanol extract. OD, optical density; N, control.

The effect of SAE treatment on lipid metabolism *in vitro* was examined by investigating its effect on the adipocyte differentiation on 3T3-L1 cells, a well-established preadipocyte cell line used for studying the conversion of preadipocytes into adipocytes. After differentiation with or without SAE, the resulting mass of accumulated intracellular lipid was stained with Oil Red O dye and quantified. As shown in Fig. 1, a dose-dependent reduction in lipid accumulation was observed in the cells treated with SAE, suggesting that SAE inhibits adipocyte transcription factors during adipocyte differentiation.

### SAE inhibits obesity in HFD-fed mice

**Figure 2:**
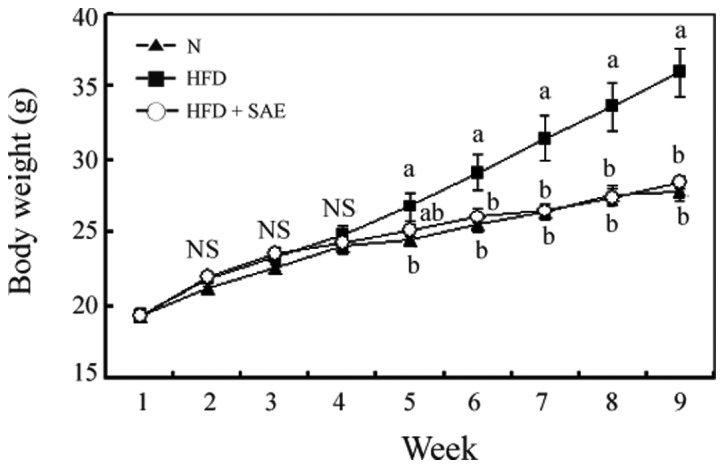
SAE reduces body weight in high-fat diet-fed mice. All the experimental animals were weighed each week. Data are expressed as mean ± SD (n=8). ^a,b^Significant differences between groups (P<0.05). SAE, *S. aromaticum* ethanol extract; N, AIN-76A diet; HFD, high-fat diet; HFD + SAE, high-fat diet + SAE supplementation. NS, not significant.

On the basis of an *in vitro* study that revealed the inhibition of adipocyte differentiation by SAE treatment, it was hypothesized that SAE may exert anti-obesity effects *in vivo*. To test this hypothesis, the body weights of the three groups of mice were compared after 9 weeks of feeding. Although no significant difference was observed between the HFD and HFD + SAE groups with respect to daily food intake, the overall body weight, as well as the liver, epididymal and retrorenal fat-pad weights, of the HFD + SAE group were found to have increased to a significantly lesser extent than that of the HFD group (P<0.05; Table I and Fig. 2), suggesting that SAE supplementation had effectively reduced fat accumulation in HFD-fed obese mice.

### SAE decreases serum TG and TC levels in HFD-fed mice

**Figure 3:**
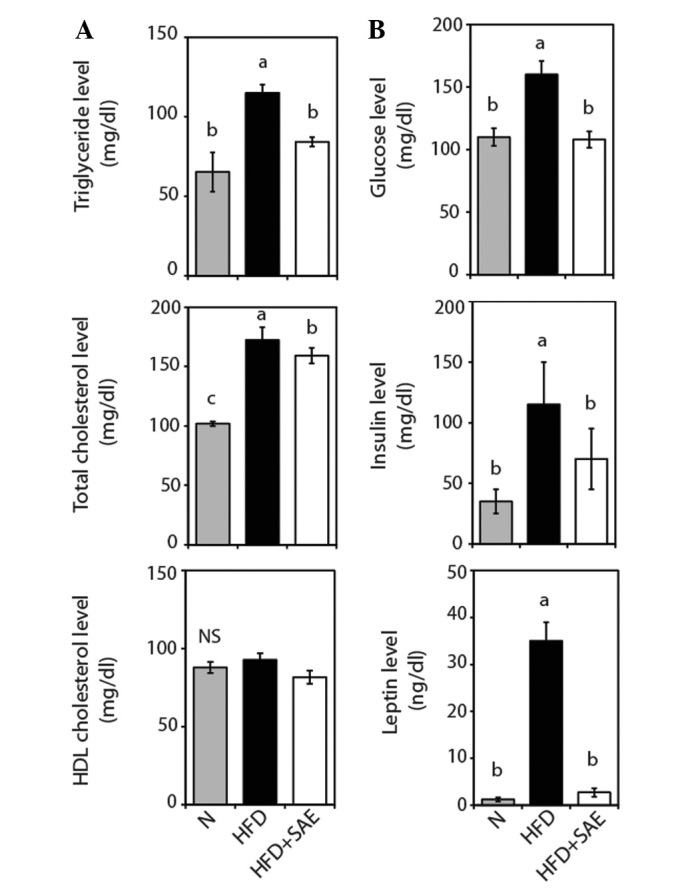
SAE improves serum tryglyceride, total cholesterol, high-density lipoprotein cholesterol, glucose, insulin and leptin levels in high-fat diet-fed mice. All the levels were analyzed using ELISA kits. Data are expressed as mean ± SD. ^a,b^Significant differences (P<0.05) in the mean values of the groups. N, AIN-76A diet; HFD, high-fat diet; HFD + SAE, high-fat diet + SAE supplementation; SAE, *S. aromaticum* ethanol extract.

After 9 weeks of treatment, the TG and TC levels of the HFD + SAE group were found to be significantly lower, 33.4 and 8.8% lower, respectively, than those of the HFD group (Fig. 3A), suggesting that SAE exerts a hypolipidemic effect in HFD-fed mice.

### SAE reduces serum glucose, insulin and leptin levels

The levels of not only serum glucose and insulin but also of leptin, an adipocyte-derived hormone, of the HFD group were found to be significantly higher than those of the normal group (Fig. 3B) and the HFD + SAE group, suggesting that the increased levels due to HFD feeding had been significantly decreased by SAE supplementation. These findings indicate that lower serum glucose and insulin levels may be due to lower insulin resistance.

### SAE suppresses adipogenesis in HFD-fed mice

**Figure 4:**
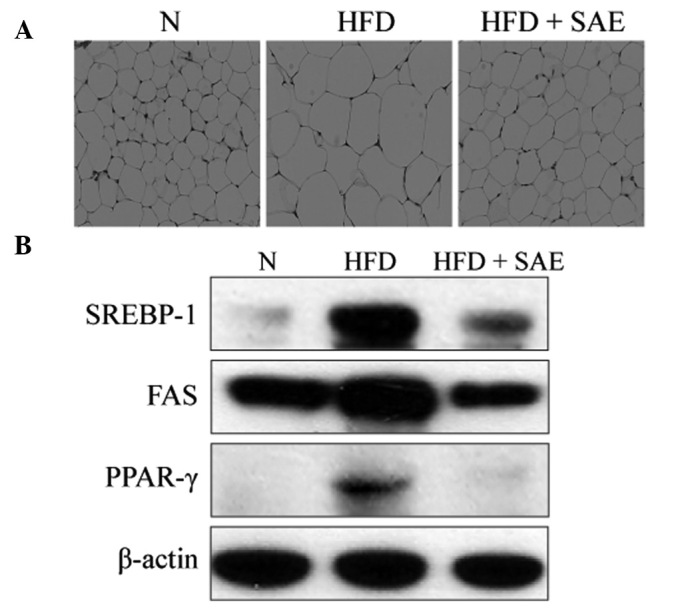
SAE reduces the size of epididymal fat pads in high-fat diet-fed mice. (A) Epididymal fat pads were fixed, sectioned and stained with hematoxylin and eosin. (B) The protein levels of adipogenic genes in the epididymal fat pads were measured by western blot analysis. N, AIN-76A diet; HFD, high-fat diet; HFD + SAE, high-fat diet + SAE supplementation; SAE, *S. aromaticum* ethanol extract.

SAE supplementation was found to have reduced the size of the epididymal fat pad in HFD-fed mice (Fig. 4A). Investigation of the manner in which SAE supplementation reduces the epididymal fat pad weight revealed that it suppresses the expression of several proteins related to adipogenesis genes, including SREBP-1, PPARγ and FAS (Fig. 4B), indicating that it exerts an anti-obesity effect by inhibiting adipogenesis.

### SAE attenuates fatty liver in HFD-fed mice

**Figure 5:**
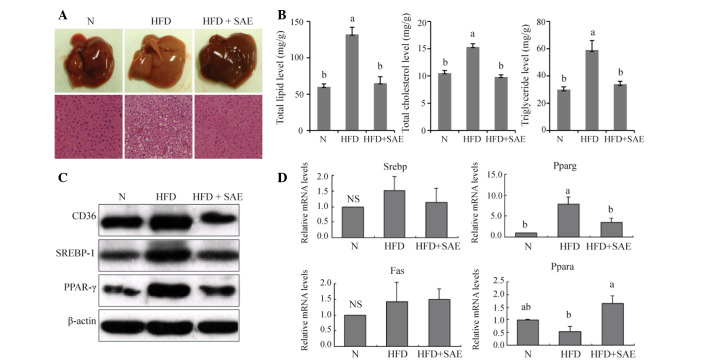
SAE regulates the protein and mRNA levels of lipogenic genes in the liver in high fat diet-fed mice. (A) Livers were fixed, sectioned and stained with hematoxylin and eosin. (B) Hepatic total lipid, triglyceride and total cholesterol levels were analyzed using ELISA kits. (C) The protein levels of lipogenic genes in the liver were measured by western blot analysis. (D) Expression of transcription factors in the liver as measured by RT-PCR. Data are expressed as mean ± SD. ^a–c^Significant differences (P<0.05) in the mean values of the groups. N, AIN-76A diet; HFD, high-fat diet; HFD + SAE, high-fat diet + SAE supplementation; SAE, *S. aromaticum* ethanol extract. NS, not significant.

Comparison of the effect of SAE supplementation on the extent of lipid accumulation in the livers of the HFD + SAE and HFD groups via H&E staining indicated reduced white coloring and fewer lipid droplets and uncolored circles in the livers of the HFD + SAE group compared with the HFD group (Fig. 5A). The total lipid, TC and TG levels in the HFD + SAE group were found to have decreased by 45.2, 37.5 and 39.5%, respectively (Fig. 5B). These results suggest that SAE supplementation had attenuated the increase in hepatic TG and TC levels due to HFD feeding.

To further explore the effect of SAE on lipogenesis, the expression of proteins and mRNA involved in lipogenesis, including that of SREBP-1 and its primary lipogenic target enzymes (e.g. FAS), as well that of PPARγ and CD36, was analyzed. The results indicated that HFD feeding of the experimental groups had resulted in significant increases in the hepatic levels of transcriptional factors integral to lipogenesis, including PPARγ, SREBP-1, FAS and CD36 (Fig. 5C). Comparison of these factors in the HFD and HFD + SAE groups indicated that SAE supplementation had reduced their levels in the HFD + SAE group, providing further evidence of the anti-lipogenic effects of SAE. Consistent with the inhibition of PPARγ by SAE supplementation, the supplementation appeared to have also downregulated the mRNA levels of Pparg (Fig. 5D), as well as to have significantly activated Ppara, a regulator of fatty acid β-oxidation. Collectively, these findings indicate that SAE supplementation inhibits the development of HFD-induced fatty liver through regulation of lipogenic genes.

## Discussion

Numerous herbs and spices have been found to reduce blood glucose levels and body weight. Among these herbs, *S. aromaticum* (family Myrtaceae) is used not only as a culinary supplement in several global cuisines but also as a traditional medicinal aid in the treatment of dental pain, headache and respiratory disorders in several Asian countries. Several studies have reported that *S. aromaticum* exerts a variety of pharmacological actions, including antioxidant, hypoglycemic and anti-inflammatory activities (7,10,11). However, to the best of our knowledge, no study has examined its action on preventing obesity. Thus, this study and its primary finding that the addition of SAE to the diet reduces body weight in HFD-fed mice, thereby indicating its potential as a natural anti-obesity supplement, are significant contributions to the research and literature in this area.

Adipocyte differentiation of 3T3-L1 preadipocytes has been found to depend on genes involved in the adipogenic pathway during the differentiation process (12). In cultured adipocyte models, SAE has been found to inhibit adipocyte differentiation in 3T3-L1 cells in a dose-dependent manner. Although analysis of the results of the *in vitro* assay performed in this study did not indicate that SAE supplementation had affected the expression of adipogenesis-related genes, analysis of the western blot results of the *in vivo* assay revealed that it had strongly suppressed an increase in WAT mass in HFD-fed mice via reduction of PPARγ and SREBP-1 expression. Analysis of these results indicates that SAE supplementation reduces WAT mass via a reduction in adipogenesis, leading to lipid loss, and thus strongly suggests that SAE supplementation prevents HFD-induced lipid accumulation in epididymal adipose tissue through regulation of adipogenesis.

HFD feeding elevates serum leptin and insulin levels, which are associated with energy expenditure, glucose metabolism and fatty acid oxidation (13). SAE supplementation was found to have significantly reduced serum leptin and insulin levels in HFD-fed mice, indicating that SAE improves these HFD-induced metabolic abnormalities. SAE supplementation was also found to have decreased HFD-induced increases in liver weight and hepatic lipid, and TC and TG levels, a finding supported by the results of H&E staining, which indicated that SAE supplementation had reduced hepatic lipid accumulation. Investigation of hepatic expression of the genes regulating lipid metabolism revealed that SAE supplementation had significantly prevented reduction of Ppara and had suppressed the expression of both the lipogenic transcription factor Pparg and lipogenic regulatory proteins, including CD36, SREBP-1, and PPARγ, thereby regulating the expression of genes promoting fatty acid oxidation. These findings suggest that SAE supplementation improves hepatic metabolic parameters via regulation of lipogenesis-related genes in the liver.

While these findings provide evidence that SAE supplementation significantly inhibits obesity in HFD-fed mice, further investigation of the active compounds responsible for anti-obesity effects is required. Kuroda *et al* recently reported that eugenol derivatives, including dehydrodieugenol and dehydrodieugenol B exert potent hypoglycemic effects via PPARγ activation in diabetic models (11). Contrary to the results of the *in vitro* assay in this study, Kuroda *et al* found that these constituents stimulate 3T3-L1 preadipocyte differentiation through PPARγ activation, indicating that unknown compounds in SAE besides dehydrodieugenol and dehydrodieugenol B may inhibit PPARγ activation and exert anti-obesity effects through regulation of adipogenic-related genes.

Collectively, the results of this study provide solid evidence that SAE supplementation exerts an anti-obesity effect on HFD-induced obese mice via regulation of genes related to lipid metabolism in the liver and WAT in a manner that results in the reduction of lipid accumulation. These results strongly support the potential of SAE as an anti-obesity supplement in the regulation of body weight.

## Figures and Tables

**Table I:** Effect of SAE on body weight, liver and WAT in mice fed a high-fat diet for 9 weeks.

Growth parameters	N	HFD	HFD + SAE
Body weight gain (g)	8.57±0.75^b^	16.60±1.72^a^	9.15±0.97^b^
Liver (g/100 bw)	3.61±0.20^c^	4.94±0.15^a^	4.08±0.07^b^
Epididymal fat pad (g/100 g bw)	1.97±0.04^b^	4.56±0.20^a^	2.03±0.15^b^
Perirenal fat pad (g/100 g bw)	0.93±0.07^b^	2.14±0.10^a^	1.05±0.14^b^
Food intake (g/day)	2.91±0.06	3.05±0.06	2.95±0.04

Values are presented as the mean ± SD (n=8).

a–c Significant difference in the mean values of the groups. SAE, *S. aromaticum* ethanol extract; WAT, white adipose tissue; bw, body weight; N, AIN-76A diet; HFD, high-fat diet; HFD + SAE, high-fat diet + SAE supplementation.
